# NF-κB in premature aging

**DOI:** 10.18632/aging.100502

**Published:** 2012-11-27

**Authors:** Fernando G. Osorio, Carlos López-Otín, José M. P. Freije

**Affiliations:** Departamento de Bioquímica y Biología Molecular, Facultad de Medicina, Instituto Universitario de Oncología, Universidad de Oviedo, 33006-Oviedo, Spain

During decades, aging has been regarded as the consequence of a stochastic process caused by the accumulative effect of damaged molecules. However, recent experimental evidences have extended this view and suggested that aging also requires active signaling programs for the maintenance of the aged state [1]. Beyond cell-autonomous alterations, age signals get systemic through changes in intercellular communication pathways [2]. The identification of the precise nature of these mechanisms and signals could provide valuable information, uncovering potential targets for rejuvenation-aimed approaches [3].

Aging research has greatly benefited from the study of progeroid syndromes, accelerated aging conditions caused by an excessive accumulation of cellular damage or by an inefficient response of the repair mechanisms. Progeroid laminopathies are accelerated aging syndromes caused by defects of the nuclear lamina. Among them, Hutchinson-Gilford Progeria Syndrome (HGPS) is one the most intensely studied. This syndrome is caused by a point mutation in the *LMNA* gene, leading to the accumulation of a truncated form of lamin A called progerin which induces important alterations in the cell nucleus. Interestingly, progerin accumulation has also been reported during normal aging, adding a new layer of interest to the study of this syndrome.

NF-κB transcription factors respond to a large variety of external and internal stress signals, having essential roles in development and tissue homeostasis maintenance. Through the study of two related mouse models of progeroid laminopathies (*Zmpste24*-deficient and *Lmna^G609G^* knock-in mice), we have recently found that aberrant activation of NF-κB is involved in the pathogenesis of accelerated aging syndromes, providing new insights into the mechanisms that allow the integration of cellular and systemic alterations in the aging process [4].

The *in vivo* monitoring of NF-κB activity by using a reporter-based assay revealed that this pathway was constitutively hyperactivated in progeroid mice. Further experiments allowed us to unveil the molecular pathway involved in this aberrant activation. Thus, in response to nuclear envelope alterations some important DNA damage sensors such as p53 or ATM were activated. In this context, we provide evidence that active ATM kinase cooperates with nuclear NEMO, an NF-κB regulatory subunit, resulting in the activation of NF-κB. We also found that, in response to NF-κB activation, several pro-inflammatory cytokines were significantly up-regulated. Among them, secretion of IL-6, CXCL-1 and TNF-α could have a causal role in the premature aging syndrome by establishing a chronic inflammatory situation through feed-forward regulatory signaling, affecting distant cells and tissues.

Aimed at dissecting the precise contribution of NF-κB hyperactivation to the progeroid phenotype, we used an anti-inflammatory genetic strategy based on crossing *Zmpste24*-deficient mice with *RelA* haploinsufficient mice [5]. Interestingly, double mutant *Zmpste24^−/−^ RelA^+/−^* mice displayed a retardation in the aging process, showing an extended longevity as compared with *Zmpste24^−/−^ RelA^+/+^* mice. Furthermore, double mutant mice showed a remarkable recovery of skin and immunological alterations, which is consistent with the proposed relevance of NF-κB activity in tissue homeostasis maintenance. These results prompted us to test a pharmacological approach to target NF-κB activation in progeroid mice. Thus, sodium salicylate treatment of both *Zmpste24^−/−^* and *Lmna^G609G/G609G^* mice extended longevity and led to a significant prevention of skin and immune alterations, demonstrating the feasibility of targeting this pathway for slowing down the progression of accelerated aging.

Finally, our results indicate that these findings can be extended to normal aging, suggesting that a common accumulation of genetic damage and nuclear envelope alterations with age could be responsible, at least in part, of the abnormal NF-κB activity reported in tissues from advanced aged donors. The accumulation of senescent cells together with the decline in adult stem cell function is a primary cause of the compromise of tissue homeostasis during aging. The primary function of NF-κB activation in this context could be related to the prevention of apoptosis of damaged cells, so that chronic activation of this pathway with the subsequent immunological decline could preclude a proper clearance of senescent and damaged cells. In this regard, a recent report has described the causal involvement of inflammatory pathways in the age-related decline in stem cell function [6].

Globally, the data discussed herein clarify three important aspects that define NF-κB role during aging. Thus, experimental data confirm that NF-κB signaling is active during normal aging [7], its hyperactivation is associated with the development of accelerated aging and its amelioration retards the aging process [4-5, 8]. These characteristics support the use of strategies aimed at controlling NF-κB related inflammation as putative rejuvenation strategies during both normal and pathological aging. Over the last years, aging research has composed a complex picture where both cell autonomous and systemic alterations cooperate for establishing the aged state in organisms. Rational design of new interventions aimed to slow down this process should act in a coordinate way, targeting pro-aging signals as well as altered cellular communication pathways for the effective prevention of aging-related disorders.

**Figure 1:**
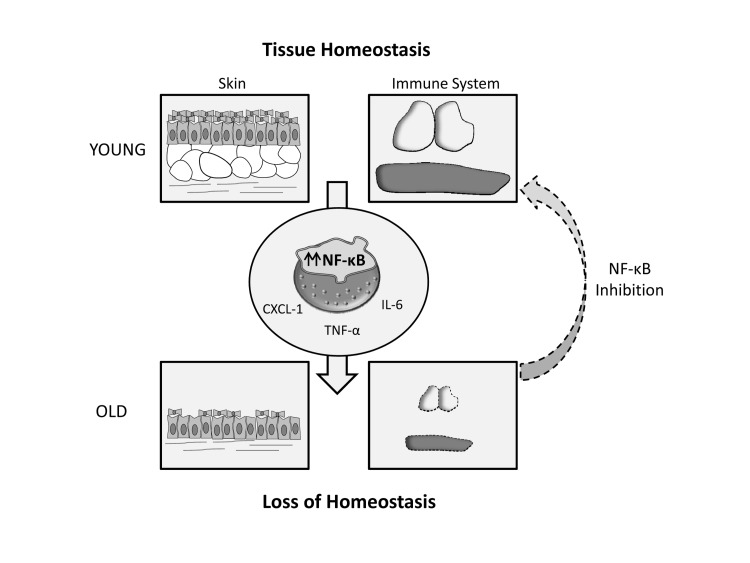
Exacerbated NF- κB signaling leads to age-related loss of tissue homeostasis, which takes place at an accelerated rate in progeroid syndromes. This phenomenon can be alleviated through NF- κB inhibition.
